# A 52-Year-Old Woman With Shortness of Breath and Left Lower Back Pain

**DOI:** 10.1016/j.chest.2024.09.035

**Published:** 2025-03-10

**Authors:** Faraz Badar, Harith Al-Ataby, Mohammed Al-Azzawi, Mohamed Omballi

**Affiliations:** Department of Pulmonary and Critical Care Medicine, The University of Toledo, Toledo, OH

## Abstract

A 52-year-old woman presented to the clinic with progressively worsening shortness of breath associated with intermittent pleuritic left lower back pain for the past 6 months. The patient denied any cough, hemoptysis, fever, chills, or weight loss. She had a history of smoking cigarettes for more than 10 years but quit almost 20 years ago. An outpatient chest radiograph was obtained, and it suggested consolidation of the left lower lobe. The patient was treated empirically with amoxicillin-clavulanate for 2 weeks without improvement.

## Physical Examination Findings

Vital signs included a temperature of 36.4 °C, heart rate of 112 beats/min, respiratory rate of 20 breaths/min, oxygen saturation of 98% on room air, BP of 122/77 mm Hg, and BMI of 24.6. On examination, the patient was in no acute distress. Chest auscultation revealed mild inspiratory and expiratory wheezing along with crackles over the left mid and lower lung zones. There was no palpable supraclavicular, cervical, or axillary lymphadenopathy. Cardiac, abdominal, neurological, and skin examinations were largely unremarkable.

## Diagnostic Studies

Complete blood count with differential was unremarkable, with WBC count of 5 K/μL, hemoglobin of 11.7 g/dL, and platelet count of 381 k/μL. Differential included 83.8% neutrophils, 5.6% lymphocytes, and 6.9% monocytes. Basic metabolic panel was within normal limits. Liver function tests were also unremarkable except for borderline elevation of alkaline phosphatase to 148 U/L (reference range, 34-104 U/L). Erythrocyte sedimentation rate was elevated to 84 mm/h. Lactate dehydrogenase was 122 U/L. Procalcitonin was negative at less than 0.05 ng/mL.

**Figure 1:**
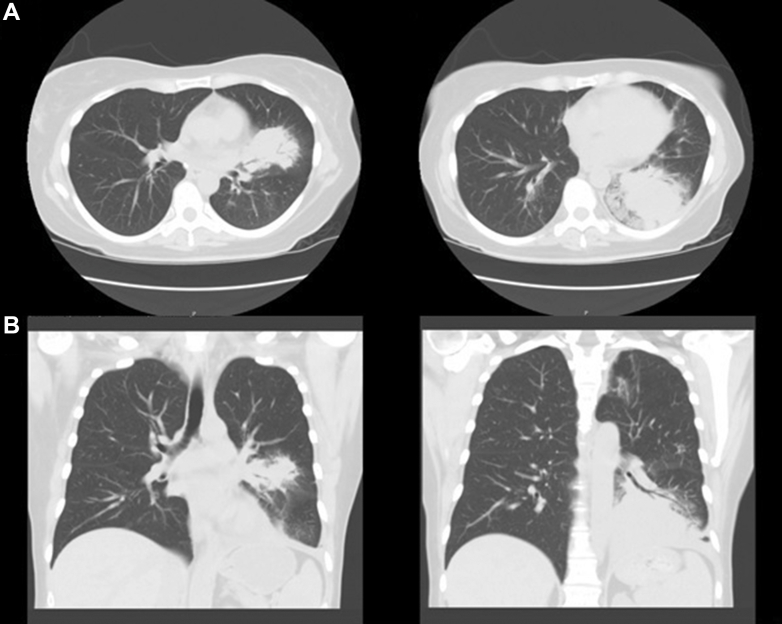
Axial (A) and coronal (B) CT imaging demonstrating ill-defined mass-like consolidations in the left upper and lower lobes.

CT scan of the chest exhibited ill-defined dense masslike consolidations in the left upper and lower lobes (Fig 1).

**Figure 2:**
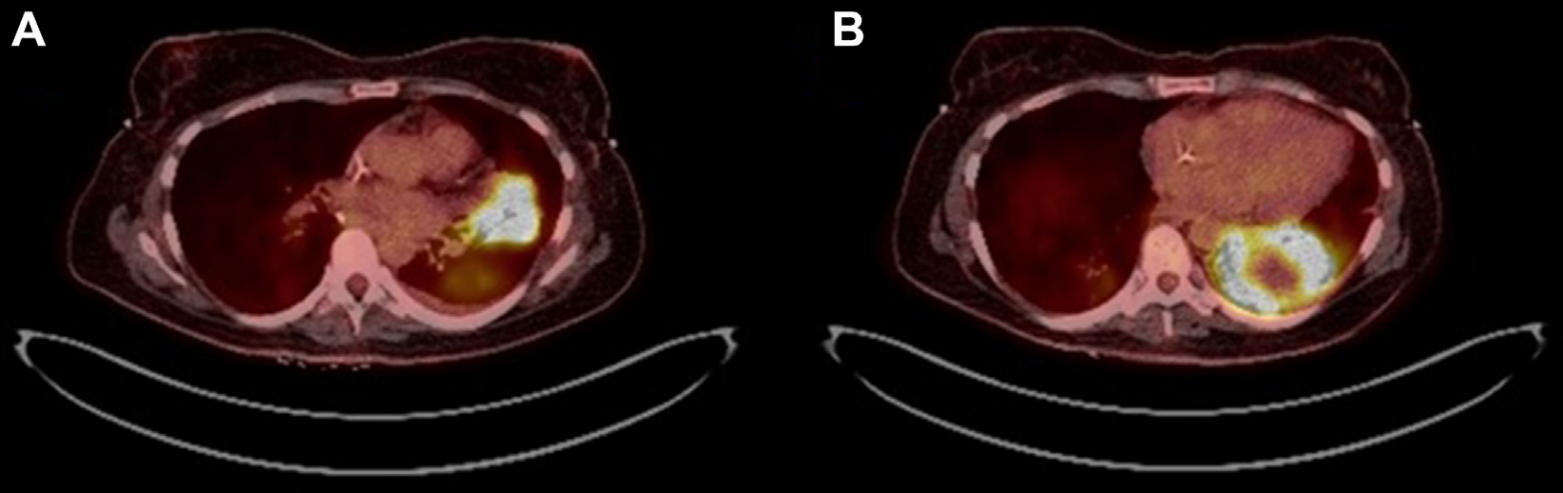
Axial PET images demonstrating left upper lobe mass measuring 4.8 × 3.9 cm with max SUV 10.6 (A) and left lower lobe mass measuring 8.7 × 5.2 cm with max SUV 13.4 (B). SUV = standardized uptake value.

PET scan showed avid consolidation in the left upper lobe measuring 4.8 × 3.9 cm with fluorodeoxyglucose standardized uptake value (SUV) of 10.6 and left lower lobe measuring 8.7 × 5.2 cm with SUV 13.4 (Fig 2). The left lower lobe consolidation was noted to have some central necrosis. Additionally, multiple PET-avid lymph nodes in the right mediastinal and hilar areas were seen. Physiologic tracer uptake was otherwise seen in the salivary glands, blood pool, liver, spleen, pancreas, bone marrow, bowel, kidneys, and urinary tract. Notably, no PET-avid lymph nodes were found in the abdomen or pelvis.

Bronchoscopy, endobronchial ultrasound guided transbronchial needle aspiration (EBUS-TBNA) and transbronchial cryobiopsy were performed. Airway inspection showed airway narrowing and mucosal thickening in the lingula and basal segments of left lower lobe. Cell count from bronchoalveolar lavage showed a WBC count of 350/μL with 26% neutrophils, 31% lymphocytes, 1% eosinophils, 27% monocytes, and 15% epithelial cells. Bacterial, fungal, and viral cultures as well as cytology from bronchoalveolar lavage were negative.

Cytology from EBUS-TBNA of the left lower lobe consolidation showed few scattered lymphocytes with no specific pathological conditions. Transbronchial cryobiopsy of the lingula and left lower lobe was performed under fluoroscopic and radial ultrasound guidance.

**Figure 3:**
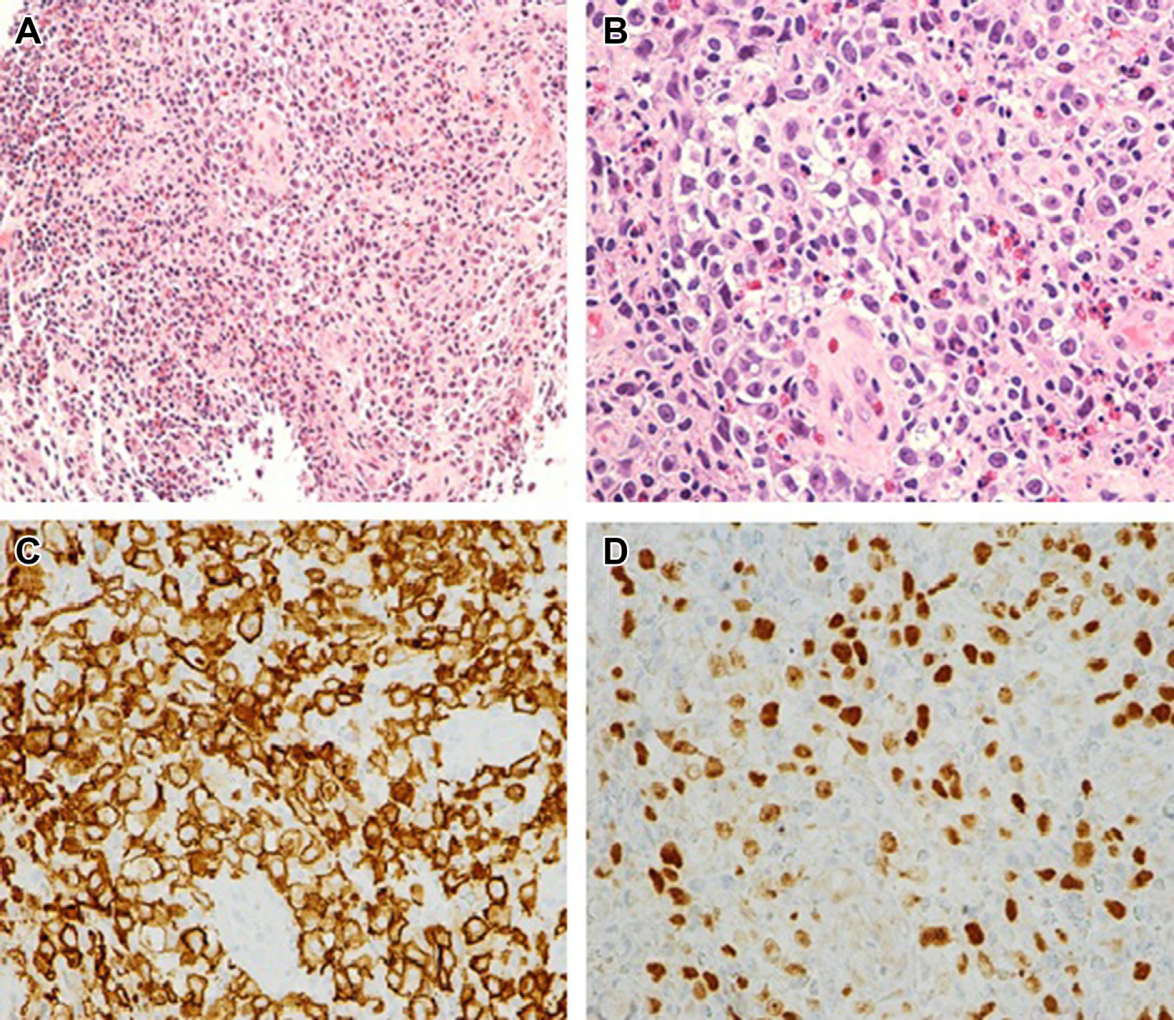
Large atypical cells in background of inflammatory cell infiltrate on 100× magnification (A) with irregular nuclear contours, large, prominent nucleoli on 400× magnification (B). Immunohistochemical analysis showing lacunar cells expressing CD30 (C) and PAX5 (D) on 400× magnification.

Histopathological examination of the cryobiopsy sample showed a polymorphous inflammatory infiltrate, consisting of numerous eosinophils, small mature-appearing lymphocytes, large multinucleated giant cells, and focally, sheets of large, atypical lymphocytes with irregular nuclear contours, large, prominent nucleoli, and rare binucleated forms. Scattered lacunar variants were also noted in these sections. Immunohistochemistry showed neoplastic cells weakly positive for CD20 compared with a background of small B cells and CD3-positive T cells. Additionally, the cells of interest were positive for CD15 and CD30 with weak staining for PAX-5. CD45 was negative in these cells (Fig 3). Acid-fast bacilli and Grocott methenamine silver special stains were negative for acid-fast organisms, pneumocystis, and other fungal elements.

*What is the diagnosis?*

*Diagnosis:* Primary pulmonary Hodgkin lymphoma (PPHL).

## Discussion

Primary pulmonary Hodgkin lymphoma (PPHL) is one of the rare, primary extranodal presentations of Hodgkin lymphoma. It constitutes less than 1% of all primary lung cancers and less than 5% of extranodal lymphomas. Patients with PPHL may be either asymptomatic or present with nonspecific signs and symptoms, such as cough, dyspnea, hemoptysis, or B-symptoms. The radiographic findings are also nonspecific and can vary from a solitary nodule, multiple ill-defined nodules, alveolar consolidation, or cavitary pulmonary lesions.

Because of the presence of equivocal clinical and radiographic findings, PPHL may mimic numerous other pulmonary pathologies, such as cryptogenic organizing pneumonia, pulmonary TB, primary bronchial carcinoma, metastatic lung nodules, fungal infections, or granulomatosis with polyangiitis. The heterogeneity of the disease and the wide array of differential diagnoses may make the diagnosis of PPHL particularly problematic and may require multiple noninvasive and invasive diagnostic tests to be performed. The criteria for diagnosis include the presence of characteristic histologic features of Hodgkin lymphoma, restriction of the disease to the lung, with or without minimal involvement of the mediastinal/hilar lymph nodes, and the exclusion of extrapulmonary disease.

Inflammatory markers, such as erythrocyte sedimentation rate, may be elevated, and patients may have anemia, leukocytosis, or neutrophilia. However, definitive diagnosis is established through biopsy. Histopathological analysis shows Reed Sternberg cells with polymorphous inflammatory infiltration. Immunohistochemistry typically shows positivity for CD15, CD30, PAX-5, and rarely CD20. The malignant cells are negative for T-cell markers.

Bronchoscopic evaluation is usually warranted in nonresolving pneumonias to exclude pulmonary pathologic conditions, such as atypical infections or malignancies. Routine cytology and transbronchial forceps biopsy are useful tests to diagnose or rule out multiple differentials.

EBUS-TBNA is frequently used for the diagnosis and staging of lung cancer. However, a review of the literature showed that it has low diagnostic yield in newly diagnosed cases of lymphoma, vs a higher yield in relapsed cases. Transbronchial cryobiopsy, which has recently gained attention as a safe and minimally invasive approach for obtaining large tissue samples, may be used.

Transbronchial cryobiopsy is a new modality that is typically used for interstitial lung disease diagnosis through sampling of lung parenchyma. It is rarely used for the diagnosis of lymphoproliferative disorders with pulmonary involvement. A recent paper involving 970 parenchymal transbronchial cryobiopsies had only 12 cases related to lymphoma, and only two of them were Hodgkin. Apart from parenchymal tissue sampling, the transbronchial cryobiopsy approach also can be used for direct sampling of mediastinal lymph nodes and lesions. A recent trial compared traditional EBUS-TBNA with transbronchial cryobiopsy of mediastinal lesions in 197 patients and revealed a higher diagnostic yield for cryobiopsy at 91.8% vs 79.9% for transbronchial needle aspiration. Cryobiopsy allows obtainment of larger tissue samples that are devoid of crush artifact. The larger sample size allows for extensive immunohistochemistry and molecular tests, whereas instant freezing does not produce significant artifacts with preservation of the normal pleural-parenchymal structures. All these factors increase diagnostic yield and help establish a pathologic diagnosis.

For treatment, combination chemotherapy is often used. No consensus exists regarding the best chemotherapy regimen for PPHL; however, doxorubicin, bleomycin, vinblastine, and dacarbazine is usually preferred. The prognosis is generally favorable, and a 5-year survival rate of 94% has been reported for patients with low-grade PPHL.

### Clinical Course

An oncology evaluation was obtained, and the patient was started on doxorubicin, bleomycin, vinblastine, and dacarbazine chemotherapy. A restaging PET scan after two cycles showed significant interval improvement in size and SUV of the left upper and lower lobe masses along with resolution of the associated lymphadenopathy. The chemotherapy regimen was later changed to rituximab, cyclophosphamide, doxorubicin, vincristine, and prednisone, and four cycles of this were given, for a total of six cycles of chemotherapy. Four-monthly surveillance PET imaging was continued, and the patient has remained disease-free at 15 months since initial diagnosis.

## Clinical Pearls

1. *PPHL is one of the very rare, primary extranodal presentations of Hodgkin lymphoma and can present with nonspecific clinical and radiographic findings.*
2. *Heterogeneity of the disease and a wide array of differentials make diagnosis difficult. Diagnostic criteria include the presence of characteristic histologic features of Hodgkin lymphoma, restriction of the disease to the lung, with or without minimal involvement of the mediastinal/hilar lymph nodes and the exclusion of extrapulmonary disease.*
3. *Transbronchial cryobiopsy permits the safe acquisition of sufficient and high-quality tissue samples from lung parenchyma, mediastinal lesions, and lymph nodes. It therefore has emerged as an important tool in the diagnosis of different diseases, including interstitial lung disease, and lymphoproliferative disorders with pulmonary involvement.*
4. *Although EBUS-TBNA enjoys widespread popularity for diagnosis and staging of lung cancer, it has a low diagnostic yield in newly diagnosed cases of lymphomas, such as PPHL, compared with relapsed cases with pulmonary involvement. Transbronchial cryobiopsy can overcome this issue and provides a higher diagnostic yield in such cases.*

## Financial/Nonfinancial Disclosure

None declared.
